# Multiraciality and mental health: the Cultural Formulation Interview as an instrument for exploring in-between identities and third spaces

**DOI:** 10.3389/fpsyt.2025.1690109

**Published:** 2025-11-07

**Authors:** Mattias Strand, Sayaka Osanami Törngren

**Affiliations:** 1 Centre for Psychiatry Research, Department of Clinical Neuroscience, Karolinska Institutet, Stockholm, Sweden; 2 Transcultural Center, Stockholm Health Care Services, Region Stockholm, Stockholm, Sweden; 3 Department of Global Political Studies, Malmö Institute for Migration Studies, Malmö University, Malmö, Sweden

**Keywords:** mental health, multiracial, multiethnic, cultural sensitivity, migration, racism, adoption, stereotypes

## Abstract

The multiracial population has grown substantially across the Global North, with Sweden having one of the largest per capita mixed populations worldwide. Despite this demographic shift, mental healthcare practitioners are often unprepared to address the complex experiences of mixed individuals, who navigate ambiguous racial and ethnic spaces that challenge traditional monolithic categories. We here include transracial adoptees as part of the mixed population, recognizing their unique experiences of racialized in-betweenness. Historical narratives have long pathologized mixed individuals through harmful stereotypes such as the “tragic mulatto” and “marginal man”, portraying them as inherently maladjusted. Contemporary research presents mixed findings regarding mental health outcomes in multiracial populations, with some studies reporting higher rates of anxiety, depression, and suicidal ideation, while others find no substantial differences compared to monoracial groups. This article argues for a more nuanced, person-centered approach to understanding mixed identity in healthcare and foregrounds the Cultural Formulation Interview (CFI) as a valuable clinical instrument. The ethnographic framework of the CFI enables exploration of complex identity negotiations, experiences of racism and discrimination, and available resources without reinforcing deficit perspectives. By fostering culturally attuned practices, mental healthcare can better serve an increasingly diverse society and address the multilayered experiences of multiracial individuals.

## 1 Introduction

Over the past decades, the multiracial and multiethnic part of the population has been steadily growing throughout large parts of the Global North. In the United States 2020 census, 10.2 percent of the population reported that they self-identify as mixed (1), having, for example, one Black and one Native American parent, one White^1^ and one Asian American parent, et cetera. This number might be an underestimation, however, since many individuals with a mixed racial background do not in fact consider themselves or choose not to identify as multiracial and report that they have been raised as one particular race (2, 3). It was only in the year 2000 that the choice to identify with multiple racial categories became available in the United States census. According to the United States 2020 census, the number of people who identify with more than one racial category changed more than all singular racial groups, increasing from 2.9 percent of the population ten years earlier (1). The mixed population is also considerably younger than the rest of the United States population, largely due to the fact that mixed-race couples have become much more common in the past few decades, and is projected to continue to grow rapidly in numbers (2).

Occasionally, these demographic projections for the future have been exaggerated and sensationalized (4). Nevertheless, similar patterns of increasing ethnic and racial diversity can undoubtedly be seen in several countries in Europe (5, 6) as well as in Australia (7) and East Asia (8, 9), although straightforward comparisons can be difficult due to the fact that different countries report different statistics and employ heterogeneous terminologies. Notably, most existing research on the well-being, representation, and social standing of multiracial and multiethnic individuals stems from the United States or the United Kingdom, where discourses on race and racism arguably tend to be more established and openly articulated than in many other contexts. This, however, should not be taken to mean that multiraciality is any less relevant in other parts of the world. For example, in Sweden—a country that maintains an officially recognized ideology of colorblindness and that does not regularly collect data on self-identified race and ethnicity (10)—around 10 percent of the population have parents who are born in two different countries (which, although far from perfect, is the closest proxy variable available for mixed race or ethnicity) (11, 12).^2^ Notably, Sweden differs from the United States and many European former colonial powers in that immigration from non-European countries is a fairly recent phenomenon. Although Sweden has a long history of systemic discrimination against the Indigenous Sámi people, Jews, Roma, and other minoritized groups based on pseudoscientific racial theories (13, 14), large-scale migration of persons of color from non-European countries only took off in the 1970s and 1980s with a growing number of people seeking asylum and family reunification (15). Nevertheless, it has been estimated that Sweden of today has one of the largest per capita mixed populations in the Global North (16). Like in many other countries, this group is projected to grow further; today, approximately 20 percent of newborn Swedes have parents who are born in different countries (11). Notably, Sweden is also home to the largest per capita population of transnational and transracial adoptees—a group that we will discuss further below as an example of a particular type of racially and ethnically based mixedness—in the world (17).

The historical, sociological, and psychological narratives and tropes about mixed race are manifold and often contradictory. Some have prophesied that a growing number of multiracial individuals will usher in a postracial society characterized by racial fluidity and tolerance. This often includes the notion that “pretty soon, everyone will be mixed” (18). Others, however, argue that the rise in multiracial identification does not necessarily alter or even problematize existing racial hierarchies and patterns of interpersonal and systemic racism—at best, it might encourage reconsiderations about how we understand race as a social category (3, 19). Another contentious issue concerns terminology. It can, for example, be argued that the use of the term “mixed race” erroneously implies the baseline existence of pure, essential races that can then be blended. Even so, when asked, those who self-identify as mixed tend to prefer “mixed race” over alternatives such as “mixed heritage” or “mixed origin”, the use of which can be seen as disregarding the real-life impact of race as a social construct (20). A third concern, addressed in more detail below, has to do with the enduring notion among the racial majority that mixed-race individuals are inherently more prone to various kinds of maladjustment. The notion that mixed-race people are somehow destined to experience feelings of being uprooted and never quite “at home” on any side of a presupposed racial divide dominated Anglo-American sociology for much of the twentieth century (21) and continues to influence psychological theories about hybridity and in-between states. For healthcare in general and psychiatry in particular, this ideological heritage raises questions about how to best attend to the mental health and well-being of patients who identify with multiple racial or ethnic categories without reinforcing harmful stereotypes.

## 2 Aim, terminology, and positionality

The aim of this “Hypothesis and Theory” article is to further our understanding of how we can better respond to the mental health needs among those who identify as mixed through clinical practice. Our contribution is threefold. First, we contextualize existing research on multiraciality to draw attention to its relative neglect in clinical discourse and to underscore its importance for culturally responsive care. Second, we argue for an expanded understanding of who is considered mixed, one that includes transracial adoptees—individuals typically raised in majority-White families and contexts yet persistently racialized as non-White in society. Third, we explore the potential usage of the Cultural Formulation Interview (CFI), included in the fifth edition of the *Diagnostic and Statistical Manual of Mental Disorders* (DSM-5) (22) as well as in the subsequent text revision, as a helpful tool in opening up a space for exploring the potentially multilayered impact of mixedness on mental health in a nuanced and person-centered manner. In this article, we use the terms “mixed” and “multiracial” to refer to individuals who identify as belonging to more than one singular racial and/or ethnic category or as existing in between categories altogether. As described above, this terminology is typically preferred by multiracial individuals when surveyed, and it is common in psychology and the social sciences (though we recognize that it may not be universally preferred across other research fields). For the sake of clarity in our discussion, we also adopt the following working definitions: *Race* refers to a socially constructed category based primarily on perceived physical differences (such as skin color) that has been used historically to organize hierarchies of power and privilege. *Ethnicity*, in turn, refers to a social category based on shared cultural traits, such as language, ancestry, traditions, or religion, emphasizing a sense of group identity and belonging. Theoretically, race tends to be an externally assigned category, whereas ethnicity is internally claimed. We readily acknowledge, however, that in practice, the boundaries between race and ethnicity is often blurred (23, 24)—not least, ethnicity (and culture, discussed further below) often serves as an implicit proxy for race in settings where the race concept is considered obsolete or taboo. In line with our second contribution outlined above—namely, the inclusion of transracial adoptees in the discussion of mixedness—we nonetheless retain the distinction between race and ethnicity. Whereas the transracial adoptee population is often treated as a separate group in research, in this article we recognize them as having experiences and affiliations similar to those of other multiracial individuals, due to the fact that they tend to identify ethnically with the adoptive family heritage but to be racialized as persons of color. Their inclusion is especially important in addressing the multilayered impact of mixedness on mental health, recognizing that the racialization of transracial adoptees and their (often) multiracial children tends to remain invisible in societal discourse (25–28).

In what follows, we begin with briefly introducing the various existing theoretical strands dealing with mixedness that exist in the literature. We then discuss the prevailing narrative of mixed individuals as an anomaly in more depth, in order to further substantiate the need for a broader and more multifaceted outlook on the mental health needs of the mixed-race population. We address the mental health of transracial adoptees in some more detail, and review the current evidence on the social and psychological well-being of mixed individuals. Last, we explore and outline the potential benefits of employing the CFI as a useful instrument in counseling and mental healthcare for individuals with a multiracial and multiethnic background. As both authors are based in Sweden, we occasionally use examples from this context to supplement the predominant focus on the United States and the United Kingdom in the existing literature. In this process, it is also our hope that our respective backgrounds in social sciences and psychiatry will be useful in creating and shaping an interdisciplinary dialogue that can contribute to a holistic understanding of the various experiences of mixed identity.

Before turning to the existing literature, we wish to situate ourselves by briefly reflecting on our positionality as researchers. MS grew up and lives in Sweden. Although his family has substantial Finnish heritage (as do many Swedes), he does not speak Finnish and has never self-identified as belonging to the officially recognized national minority of Sweden Finns. He is the father of two mixed-race children and self-identifies as male, cisgender, and White—although in a Swedish majority-White context, this seldom amounts to a marked or meaningful social position. SOT grew up in Japan with an in-between identity, and was often labeled as “not Japanese” due to being bilingual (Japanese and English) and having had transnational experiences outside the country. As a young adult, she immigrated to Sweden, which she now calls home, while Japan has become a place she visits. She has spent half of her life in an interracial relationship and is raising two mixed-race children. She self-identifies as Asian, Japanese, cisgender female, and a first-generation immigrant.

## 3 Mixed and in-between identities in the literature

A nuanced understanding of the scholarship on mixedness and in-betweenness is essential for appreciating the complex social and cultural dynamics that can shape clinical experiences. A number of theories that account for the various states of in-betweenness experienced by mixed-race individuals and others exist in the literature, often grounded in postcolonial scholarship. These include the *third space* envisioned by Homi K Bhabha (29), various takes on Caribbeanness and the creole (30–33), the *new ethnicities* theorized by Stuart Hall and others (34, 35), and so on. A seminal contribution to this literature is Chicana scholar Gloria Anzaldúa’s *Borderlands/La Frontera: The New Mestiza*, originally published in 1987 (36). Here, Anzaldúa address a growing population in the South Central and Southwestern United States that exists on the inherently hybrid borderlands between geographical, ethnic, and gendered life-worlds, theorizing a mestiza consciousness that rejects a dualistic understanding of cultural identity. Common to most of these accounts is an emphasis on mixedness as a distinct experience that exists beyond simplistic views of hybridity as a salad bowl of discrete identities. This notion is echoed in the broader scholarship on intersectionality initiated by Kimberlé Crenshaw (37), by which various sociocultural hierarchies interact in ways that defy a straightforward “1 + 1 = 2” logic. Notably, the intersections of mixedness, gender, class, and place have increasingly been emphasized in the literature (38, 39).

The 1990s saw the emergence of *critical mixed race studies* as an academic field. In an influential text titled “Bill of Rights for People of Mixed Heritage”, originally written in 1993 and later expanded upon, psychologist Maria Root outlined a set of affirmations for mixed-race individuals as a reminder that they need not be confined by other’s preconceived notions of race and racial ambiguity (40). At the same time, questions were being raised about the extent to which mixed-race individuals have something in common on an experiential level—in spite of the large heterogeneity within the group—due to their shared familiarity with what might be called third or hybrid spaces. There are substantial differences in how this in-betweenness is experienced across various subgroups. For instance, a large share of individuals with African American heritage in the United States also have White and Native American ancestry, and nevertheless view themselves—and are viewed by others—as Black, first and foremost. In contrast, mixed-race Asian American and White or Native American and White adults in the United States tend to feel that they have more in common with the White population (2, 3). Socioeconomic status can also affect mixed-race identification. For example, a resourceful middle class might more readily have access to a chic *world citizen* version of mixed-race identity, whereas poorer marginalized groups might not (39). Also, individuals who are raised as White in predominantly White middle-class neighborhoods might come to identify as mixed and/or Black (or become conscious of being treated as members of a racially visible group) when they subsequently move to another place, such as when attending college (41). As touched upon above, preferences also differ when it comes to terminology. For some, viewing oneself as “multiethnic”—identifying, for example, as “Jamaican Scottish” or “Korean and Filipino”—rather than multiracial is more relevant (4, 42). For others, ethnicity is less meaningful than race:

“That’s really neat, that you can draw upon two different heritages.” I am never really sure what that means since both of my parents are pretty distant from any ethnic roots. Beyond being “culturally” American and “biologically” biracial, I cannot figure out how to access those German and Japanese heritages that people keep talking about! (18, p. 5).

Regardless of personal preferences in terms of racial, ethnic, or cultural frameworks, the salience of the various components that make up one’s identity is very often fluid, situation dependent, and subject to change over time, a tendency that might be particularly distinct for those with mixed racial heritage. This can be a liberating experience; it has been suggested, for example, that mixed-race individuals might find it easier to question other, non-racial boundaries such as gender roles or sexual preferences (42). On the other hand, an idealized view of mixed race as emblematic of postmodern ambiguity can feel overly theoretical and “abstracted from the local ground in which one lives one’s presumably decentered life” (43, p. 71). In response to such tendencies, critical accounts of mixedness have increasingly explored a negotiated *hybridity-of-the-everyday* (44), grounded in lived experience and local knowledge.

Although a critical mixed race discourse in academia only emerged in the 1990s, there is a long history of thinking and writing about mixed and in-between identities. In literary fiction and autobiographical work, lived experiences of mixedness have been a recurrent theme at least since the Harlem Renaissance of the 1920s. In works such as Nella Larsen’s *Quicksand* and *Passing*, James McBride’s *The Color of Water*, John Agard’s *Half-Caste*, and, more recently, Michelle Zauner’s *Crying in H Mart*, mixed and/or hybrid racial and ethnic identities are key motifs.

Thus far, we have mainly dealt with mixedness in a North American and European context; admittedly, our account will mostly focus on the Global North. Of course, throughout colonial history, mixed-race populations have emerged as a result of intermarriage and sexual encounters between colonizers of European ancestry and Indigenous peoples, between slave owners and enslaved individuals, and so on (45–47). In many instances, these encounters involved sexual violence—including rape—perpetrated by colonial or slave-owning elites. Various systems of indentured servitude have also contributed to mixed-race populations across the globe, such as Indo-Caribbean and Sino-Malaysian groups. As described in more detail by Francisco Bethencourt and others, this very existence of mixed individuals has often come to involve intricate caste-like racial hierarchies based on skin color and bloodlines (48–51), with, for example, terms such as *quadroon* and *octoroon* used to designate individuals with one-quarter and one-eight Black or Indigenous ancestry. In some parts of the world, mixed racial heritage is more or less the norm; for instance, it can be argued that modern Mexico and other countries in Latin America are built on an ideology of mixedness or *mestizaje* that is sometimes used to gloss over the continued existence of racism (52, 53).

## 4 From the one-drop rule to the tragic mulatto

Understanding the historical context of multiraciality is essential for clinicians, as these legacies continue to shape social attitudes and influence the lived experiences and well-being of multiracial patients and clients. There is undoubtedly a long tradition of mistrust, discrimination, and racism towards mixed-race people. Throughout the modern history of White supremacy, the existence of mixed-race individuals has been regarded as an imminent threat to Whiteness. In the United States, various state-level versions of the infamous *one-drop rule* regulated whether a mixed person would be treated as Black or allowed entry into the realm of White respectability, the underlying idea being that having even one ancestor of African heritage would render an individual Black. Similar legislation based on blood quantum was introduced in the Indian Reorganization Act of 1934, which mandated that Native American tribal status and belonging should be determined by degree of blood—an idea previously unheard of among tribal communities, where mixed heritage was common. In colonial-era Germany, intermarriage between African or Southeast Asian colonial subjects and White Germans was seen as a threat to political order; in a subsequent context of Nazi *Volksgemeinschaft*, it amounted to racial “pollution” and forced sterilization of mixed-race children was carried out in the Rhineland and elsewhere (54). Turning to Sweden, where legal sanctions against miscegenation have never officially existed, a widespread fear of the Roma population mixing with White Swedes was a key factor in the establishment of the State Institute for Racial Biology in the early 1920s (55, 56). Among White supremacists, an emphasis on miscegenation as a threat to racial purity and on White motherhood as a racial obligation in order to prevent the dilution of Whiteness has lived on (57). Notably, however, these ideas are not necessarily a fringe phenomenon. Much of the European New Right and the so-called cultural turn in racism is built on a notion of *ethnopluralism*, implicating that different cultures—used here as a euphemism for races and ethnicities—should be respected in their uniqueness but that they can and should not coexist or mix (58).

The emergence of mixed-race groups has also long been described as problematic in the fields of anthropology and sociology, although not necessarily in explicitly racist terms. In the 1920s and 1930s, British scientists took an interest in the fairly substantial mixed Chinese-White or Anglo-Caribbean communities inhabiting the docklands of British seaports, their reports often (but not always) shrouded in a language of moral condemnation (59). During the interwar period, sociologists typically depicted colonial and racial conflict as emanating from a failure on the part of colonial subjects or racialized groups to adapt to Western/White modernity (21); thus, a dominant narrative of *maladjustment* was born, of which mixed people in particular became emblematic. In a couple of seminal papers, the mixed-race individual was described as a “marginal man”, lost to identity confusion and misdirected bitterness, a stranger to himself and an outsider to the communities of both of his parents, destined for a never-ending oscillation between racial superiority and inferiority (60, 61). After scientific racism had become discredited in the wake of the Second World War, the maladjustment trope still lived on in the popular imagination. For example, the stereotype of *the tragic mulatto*—prone to self-loathing, substance abuse, and suicide attempts; often despising Black people along with the Blackness in herself—has continued to plague cinematic and literary portrayals of mixed-race people (62). In a somewhat ironic turn, race as a biological entity is nowadays sometimes invoked in largely unsubstantiated claims that racial outmarriage yields genetically-based *advantages* for the offspring, so that mixed-race individuals are supposedly more successful and rated as more attractive (4).

Parenting children with a mixed racial background has also often been depicted as inherently difficult and demanding, based on the maladjustment narrative described above. For example, preparing children for navigating racialized landscapes, including dealing with racism and discrimination, might hypothetically prove to be complicated in a mixed-race family. In reality, however, for some families it could also very well turn out to be a more straightforward task, given that mixedness can provide an intuitive platform for these discussions. Similar to patterns of subjective mixed-race identification, available parenting approaches might differ depending on socioeconomic status and class background. It has been suggested that a highly individualized, post-racial, and cosmopolitan outlook on children’s mixedness *or*, in contrast, an explicit embracement of mixed-race heritage and identification as valuable in and by itself may both be more common in middle-class environments, whereas fostering a commitment to one singular heritage is supposedly a more readily acceptable approach for socioeconomically disadvantaged families (39). Within this framework, encouraging children’s free exploration of racial identity is something that the middle class can afford, while parents from less affluent backgrounds more often feel a need to protect their children from stigma by adhering to dominant, non-mixed racial schemata. Even so, these patterns are probably also heavily influenced by the specific racial identities of parents, neighborhood characteristics, and so on. For example, “free” exploration in a White middle-class environment might often mean that mixed-race children simply grow up identifying as White, first and foremost (41), whereas for children and adolescents in Black and/or African American neighborhoods, being mixed *and* Black is often a more readily available option (2, 3).

## 5 The mental health of multiracial individuals

Population-level research on psychological and mental health outcomes among multiracial people has mostly been carried out in non-clinical populations. In brief, two opposing trends emerge from the literature. Some studies report no substantial differences in terms of socioemotional well-being between mixed and monoracial groups (63–66); occasionally, this research explicitly positions itself against a prevailing “deficit perspective” that tends to view a non-mixed experience as the “normal” state of being (64). In contrast, another larger body of literature points to a higher prevalence of various types of mental health pathology—such as anxiety, depression, posttraumatic stress, self-injurious behaviors, suicidal ideation, and suicide attempts—in mixed-race individuals as compared to the non-mixed population (67–73). Higher rates of health risk behaviors such as tobacco and alcohol use among mixed adolescents compared to their monoracial peers have also been reported (74). Notably, a subset of this literature specifically highlights racism and racial discrimination as key predictors of mental health outcomes in the mixed population (67–69). For instance, a mixed-methods study conducted among individuals identifying as multiracial and multiethnic reveals that negative mental health outcomes were significantly correlated with exposure to trauma, discrimination, and microaggressions, whereas secure identity formation and community support emerged as protective factors (67). In the same study, qualitative interviews further illuminate the unique challenges that mixed individuals face in navigating and accessing mental healthcare, underscoring the need for a more nuanced understanding of intersectional identities and cumulative traumatic experiences specific to this population.

There are, however, substantial methodological limitations that hamper the generalizability of these findings. For example, authors who have performed systematic and scoping reviews in the field note that most available research on the mental health of multiracial individuals has been carried out in the United States (69) using samples consisting of high school or college students (68). Perhaps most importantly, the common use of a single multiracial category in epidemiological research, by which all individuals who self-identify as mixed are treated as a monolithic entity, disregards the complex heterogeneity within this population and risks obscuring within-group differences (68, 69, 75, 76). It is, for example, plausible that mixed individuals who self-identify as belonging to two or more non-White marginalized racial groups (i.e., Black and Native American) are more severely affected by racial health disparities than mixed individuals who partly identify as White (68). On the other hand, a study that does include both aggregated and disaggregated data on various mixed groups shows that the multiracial population as a whole displays *better* mental health outcomes than the non-mixed comparison sample; however, when considering mixed subgroups, it turns out that those that identify as both White and non-White rated their mental health as worse compared to non-mixed persons and persons identifying as belonging to two non-White groups (77). The within-group dynamics of the mixed-race population are clearly complex. In one study, multiracial individuals who identify as such display higher levels of psychological well-being, self-esteem, and social engagement than multiracial individuals who identify with only one racial group (78). However, other research indicates that these positive outcomes cannot be observed when individuals experience their multiracial identities as highly separate or as being in tension (79). Similarly, individuals with a mixed minoritized and White racial background who experience constraints in or denial of their White identity report elevated levels of stress and depressive symptoms (80, 81). Furthermore, research examining substance use and alcohol consumption among multiracial young adults finds that individuals who report higher levels of racial ambiguity (as well as lower self-esteem and elevated depressive symptoms) are more likely to engage in drinking and substance use (82). However, this study also identifies strong familial bonds—particularly with primary caregivers—as a protective factor, significantly reducing the likelihood of substance use.

Factors such as parental gender, social capital, and social belonging also matter. A study utilizing data from a sample of United States adolescents in grades 7 through 12 compares emotional and social well-being across White, monoracial minoritized (four groups), and multiracial (four groups) adolescents (83). The findings reveal notable variations in well-being between multiracial groups and their monoracial counterparts; in addition, they show that not only race but also the parental gender configuration plays a significant role. Specifically, multiracial adolescents with a minoritized mother exhibits a distinct disadvantage in terms of psychological well-being. Additionally, higher levels of social capital are associated with reduced negative effects related to both race and parental gender configuration, suggesting its protective role in adolescent well-being. Similarly, social belonging accounts for significant variance in reported well-being in another study (80).

Turning briefly to the Swedish context, it is noteworthy that, although the country has a relatively large mixed population, little is known about their mental health and well-being. The few existing quantitative studies based on national datasets indicate that persons of mixed origin may experience different kinds of disadvantage, discrimination, and racism in Sweden; however, these outcomes have not been analyzed in connection to psychological well-being (84–86). Insight into how mixed individuals negotiate the complexities of identity-related issues is also scarce in Sweden. Sayaka Osanami Törngren’s qualitative interview-based studies (87, 88) are, to this day, the only academic research with a specific focus on how individuals with mixed racial or ethnic backgrounds navigate identity in the Swedish context, where national belonging is often implicitly tied to Whiteness.^3^ Although many mixed Swedish interviewees articulate fluid and multifaceted identities, they frequently experience identity incongruities and often encounter rigid societal expectations by which they are categorized as either Swedish or non-Swedish. This binary framing tends to exclude those who do not conform to the racial norms of Whiteness, leading to experiences of identity invalidation. Osanami Törngren’s work clearly shows how *racial ascription*—i.e., whether one is perceived by others as White, Black, Latino, Asian, or Arab—shapes the extent to which mixed individuals are accepted as Swedish. Interviewees underscore the emotional strain of having their identities questioned and describe a multitude of strategies that they employ to assert their belonging, including emphasis on mixed heritage and identity. These findings emphasize the profound impact of external invalidation on internal identity processes and emotional well-being.

## 6 The particular mixedness of transracial adoptees

So far we have focused on lineage-based mixedness—i.e., having birth parents of differing racial or ethnic identification and affiliation. In this article, however, we also include transracial adoptees as part of the larger mixed population instead of treating them as a separate group, in order to address and draw from their experiences. While dominant narratives often conceptualize mixed-race identities through blood-based frameworks (as outlined above), we wish to challenge such biologically reductive models, and instead emphasize the sociocultural dimensions of mixedness so as not to render invisible the mixed families in which transracial adoptees are socialized. Transracial adoptees—i.e., for all practical purposes, adoptees of color who are raised by White adoptive parents—often experience a particular kind of in-betweenness in terms of race and ethnicity, a distinct experience that nevertheless have certain commonalities with that of other mixed individuals. In many parts of the world, transnational adoption of children born in the Global South to (predominantly) White parents in the Global North has dominated, whereas in the United States, domestic transracial adoption of children of color has also been a contested phenomenon (89). Moreover, forced domestic adoption of Indigenous children into White families has occurred in many settings. Perhaps the most well-documented example is the coercive assimilation of Native American and First Nations children into the dominant Euro-American or Euro-Canadian culture, mainly through the widespread use of residential schools (90, 91)—where Indigenous languages and practices were prohibited and physical, emotional, and sexual maltreatment was pervasive—but also through adoption (92). Similar examples exist of Greenlandic Inuit children being adopted into Danish families for pseudoscientific purposes in the 1950s (93). In addition to exposing Indigenous children to abuse and cultural erasure, these policies fostered a conflicted state of in-betweenness, shaping complex patterns of identity and belonging that continue to affect Indigenous communities.

Transnational adoptees in particular can typically describe having been raised in a largely colorblind family environment, with a lack of serious efforts on the part of the adoptive parents to create a natural connection to one’s country of birth (25, 94, 95)—indeed, this has often been a semi-official recommendation in order to avoid “identity confusion”. For many, this can lead to a certain experience of mixedness, shaped by the fact—again using Sweden as an example—that they are socialized within their predominantly White families as ethnically Swedish and yet come to be racialized as persons of color and, consequently, not-quite-Swedish in interactions outside the family. Unlike children of immigrants who may also erroneously be treated as “foreigners” in a majority-White society, transnational adoptees usually cannot rely on a sense of shared belonging within the family in relation to one’s racial and ethnic background. Whereas, for example, members of the Ethiopian migrant community in Sweden might be able to find solace and support in a sense of communal pride in their heritage in the face of racism and discrimination, this option is usually not readily available for a transnational adoptee born in Ethiopia who has had little contact with a broader Ethiopian diaspora or with other Black people in general. (Conceivably, the experiences of groups such as asylum-seeking unaccompanied minors residing in White foster families may, in some respects, mirror those of transracial adoptees, yet they typically retain a salient connection to their country of origin).

It is nowadays well-established that transracial and transnational adoptees exhibit a markedly increased risk of various negative mental health outcomes. Compared to the rest of the population, transnational adoptees exhibit an increased risk of psychotic disorders (96), substance use (97), and disordered eating (98). Moreover, they display more symptoms of attention-deficit/hyperactivity-disorder (99) and are more likely to be in specialist outpatient and inpatient psychiatric treatment (100–102). The most alarming finding is perhaps that transnational adoptees are three to four times more likely to make a suicide attempt and to commit suicide compared to the population at large (100, 103, 104). A long list of biological, environmental, and societal factors has been suggested to explain these disparities. Recent research has tended to focus on the contributing impact of post-adoption factors such as racism and colorblindness, which might leave transnational adoptees having to navigate racialized stereotypes on their own without much helpful support from their adoptive families or from the society at large (25, 105–109). Importantly, similar to popular images of mixed-race persons, there is a prevailing notion of adoptees as “psychologically damaged” by default that might ultimately hinder effective treatment interventions (110, 111).

## 7 Exploring mixed identities with the Cultural Formulation Interview

Our synthesis of the literature reveals notable difficulties in capturing and addressing the complexities of mixed-identity experiences in relation to mental health. This knowledge gap highlights the potential value of employing person-centered, culturally sensitive tools—such as the CFI, which has been described as a “mini-ethnography”—to better capture the lived realities of multiracial individuals (112).

The CFI, included in the DSM-5 (22) as well as in the subsequent text revision, is a person-centered instrument for systematical appraisal of the impact of cultural factors on the clinical encounter. Cultural factors, as used here, refer to elements of a group’s intergenerationally transmitted heritage that shape individuals’ beliefs, values, practices, and interactions, including norms, traditions, language, religion, social structures, and shared behaviors that influence how people perceive, interpret, and respond to the world. The core component of the CFI comprises 16 open-ended questions related to the respondent’s cultural understanding of health and illness; sociocultural identity, stressors, and resources; cultural aspects of coping and help-seeking; and the patient-clinician relationship. There are also 12 supplementary modules that can be used to address topics relevant for specific populations, such as children and adolescents, the elderly, or migrants and refugees. The CFI has proven to be a feasible, acceptable, and useful instrument for exploring cultural context and identifying treatment barriers in various clinical settings (113–115). Not least, the CFI has been shown to enhance patient rapport through increased satisfaction associated with the person-centeredness of the clinical interview (116). The CFI has also been found to improve diagnostic accuracy by providing contextual information that may, for example, clarify what does and does not constitute psychotic delusions within a given cultural framework (117, 118). In response to doubts concerning limited feasibility in a busy clinical context, it has been argued that the CFI can be completed by a trained clinician in around 25 minutes (113). It is, however, our experience that the interview as a whole and some questions in particular—such as those addressing cultural identity and its impact on health and illness—often benefit from a somewhat less hurried approach, allowing for repeated consideration over several sessions. This might be particularly helpful for clients and patients who navigate ambiguous identities or who are used to providing fixed, simplified answers as a safeguard against tedious and intruding questions about race and ethnicity.

The creation and establishment of the CFI can be seen as part of a growing recognition within psychiatry of the ways in which cultural differences between patients and clinicians can create barriers when it comes to help seeking, patient-clinician communication, and assessment. Differences in terms of race and ethnicity as well as gender and gender identity, sexual orientation, age, language, socioeconomic status, educational level and vocational background, religious beliefs, and/or disability, etc. may contribute to misunderstandings, lack of insight by clinicians into a patient’s lived reality, and failure to establish rapport and trust. Failure to recognize and address any such barriers can ultimately result in poor treatment alliance and prolonged illness. Importantly, as touched upon above, the CFI was originally developed as a sort of “mini-ethnography” (112) within an epistemological framework heavily influenced by medical anthropology, which emphasizes the immersion of the researcher-clinician in the local world of the patient. From this perspective, the CFI becomes an instrument for elucidating *emic* or “insider” knowledge regarding culture- and context-sensitive illness explanatory models that may often challenge existing biomedical models. Some researchers in the field therefore caution against a popular view of the CFI as being merely a clinical “tool” for assessment that upholds an *etic* from-the-outside-looking-in perspective on the client or patient narrative (119). A more genuinely ethnographic approach to the CFI would involve a component of self-reflexivity by which the patient’s story might actually become a vehicle for furthering the clinician’s understanding of herself and the things that are taken for granted within her own sociocultural milieu, rather than a supposedly neutral and disinterested assessment tool. Thus understood, the CFI also becomes an aid in translating social science for clinical purposes (120). It is our belief that these characteristics make the CFI a particularly helpful instrument in exploring and addressing the in-between identities and third spaces that often constitute mixed individuals’ lived realities—realities that are inevitably shaped by pre-existing, monolithic categories and stereotypes that require a reflexive approach and moving beyond merely “collecting patient meanings” (121, p. 555).

Before we discuss how the CFI can be used in working with mixed clients and patients, we also wish to briefly acknowledge the ongoing academic debate concerning the potential shortcomings of the CFI (and, by extension, cultural psychiatry at large) in identifying and addressing social determinants of health, including patients’ access to various types of socioeconomic resources (122). A complementary focus on “upstream”, *structural* aspects of health and illness—including, for example, factors such as public policy, neighborhood environment, zoning laws, housing, food security, employment opportunities, racism, and discrimination—has therefore been suggested (123). Others, however, hold that the demarcation between cultural and structural facets of relevance to healthcare is typically vague and poorly defined (124). In what can perhaps be viewed as a parallel criticism, it has been argued that the notion of culture in the DSM-5 is overly focused on *meaning* at the expense of *practice*, which makes the manual “good at dealing with ideas, but [ … ] bad at grasping material, nonhuman things that co-constitute everything that humans experience” (125, p. 143). While a more detailed account of these problems is beyond the scope of this article, we note that the issues that often affect the mental health of multiracial individuals tend to involve a variety of components that can be categorized as both cultural (i.e., societal tropes and stereotypes, language and code-switching, and negotiations around cultural belonging) and structural (i.e., racism and discrimination).

Notably, a small-scale pilot study on the use of the CFI in Swedish specialist eating disorder treatment has shown that the instrument can be helpful in opening up a space for exploring the impact of patient experiences of in-betweenness with multiracial individuals, transnational adoptees, and children of immigrants (126). Not least, the participants specifically highlight the potential of the CFI to initiate and facilitate discussions about the impact of racism on their health and well-being. These findings warrant further discussion about the CFI as a potentially useful instrument in attending to experiences of interpersonal, structural, and internalized racism as experienced by multiracial clients and patients, in society at large as well as in healthcare settings. Exposure to racism is widely recognized as contributing to negative mental health outcomes such as anxiety, depression, psychosis, and substance use (127, 128), possibly mediated by an increase in allostatic load and by cumulative effects of so-called *weathering* (129). Moreover, structural racism has profound negative impact on many social determinants of mental health, such as household income, employment, education, housing and food security, neighborhood characteristics, etc. (130).While preferred terms may change across time and context, mental health practitioners need tools for facilitating critical discussions about how race informs their practice and how racial disparities in health impact their clients and patients (131, 132). For example, mixed individuals often face racial discrimination of a kind that differs from that encountered by monoracial minoritized peers. Acknowledging this, Marc Johnston-Guerrero and Kevin Nadal have developed a taxonomy of microaggressions specific to multiracial individuals, which encompasses five central themes: exclusion and isolation, exoticization and objectification, assumption of a monoracial identity, denial of multiracial reality, and pathologizing multiracial identity and experiences (133). The CFI can potentially facilitate further exploration of the impact of these themes in the lives of mixed clients and patients.

In our experience, a key strength of the CFI lies in its ability to enable nuanced exploration of experiences that have previously remained unspoken or overlooked. It is not uncommon for clients and patients to respond to the CFI with reflections such as: “Why has no one ever asked me these questions before?” For mixed individuals, this may be a particularly novel and illuminating experience. Laura Reid Marks and colleagues argue that, as agents of social justice, mental health practitioners have a responsibility to advocate for multiracial individuals by increasing their visibility across research, clinical practice, and public engagement initiatives (134). To this end, they emphasize the importance of equipping therapists with instruments and frameworks for actively engaging with the lived realities of multiracial clients and patients. Through the use of targeted questions that explore the sources and contexts of discriminatory messages—e.g., “What messages do you receive from your friends about your racial identity?”, “Which family members do you feel accepted/not accepted by due to your racial background?”, and “How are these messages communicated to you?” (p. 322)—multiracial individuals can be supported in recognizing the value of integrating their multiple racial identities, fostering greater self-acceptance and psychological resilience. Questions like these stand in stark contrast to the topics mixed individuals report being routinely asked about by healthcare practitioners, acquaintances, and even strangers, such as personal details about their parents’ relationship or pressure to choose a single racial identity (95, 135). These areas of inquiry align well with and can be embedded within the existing CFI framework.

Importantly, we also believe that the approach behind the CFI can be useful in elucidating clients’ and patients’ lived experiences without adhering to established narratives about mixed individuals as inherently maladjusted. Unlike many other clinical assessment instruments, the CFI explicitly explores available supports and resources—particularly within some of the 12 supplementary modules—although, admittedly, aspects of resourcefulness related to identity formation could benefit from an even greater emphasis. Several scholars have identified psychological strategies that enhance resilience among multiracial individuals. One such strategy is the ability to fluidly shift between multiple racial identities depending on context. This phenomenon is often described as racial *code-switching* (136); a situational and strategic (although not necessarily fully deliberate) way of navigating a racialized world by which individuals adjust how they express their racial identity based on social context. Although negative aspects of code-switching—such as fatigue linked to the demands of emotional labor, potential accusations of “acting White”, and the risk of masking symptoms of psychological distress (67, 137)—are sometimes emphasized, an ability to draw on and transition between various facets of one’s identity can also foster a sense of agency. Another potential resource associated with mixedness is the rejection of essentialist notions of race, allowing individuals to construct self-defined, nuanced, and flexible understandings of their racial and ethnic identity (75, 76, 138). Importantly, this capacity is not undermined by a simultaneous variability or inconsistency in how mixed individuals identify and code-switch across contexts. Research challenges the assumption that identity incongruence necessarily reflects an internal struggle to establish a coherent sense of self, and instead points to the role of external validation in shaping psychological outcomes (65). Regardless of the precise role that code-switching and non-essentialist notions of identity play in a client’s or patient’s life, the CFI can be a helpful instrument in systematically exploring and addressing these topics.

Here, it should be acknowledged that a few multi-item scales specifically designed to assess various dimensions of mixedness do exist. For example, the Multiracial Experiences Measure (139) includes five subscales related to shifting expressions, perceived racial ambiguity, creating a third space, multicultural engagement, and multiracial discrimination. Furthermore, the Multiracial Youth Socialization Scale (140) is a 62-item tool with eight subscales designed to assess how multiracial youth are socialized by their primary caregivers. In clinical settings, practitioners can potentially use this scale to identify the types of racial messages that young people receive, address potentially harmful messages, and support families in fostering more affirming and inclusive communication around race. Even so, these scales are primarily designed for research purposes, initial clinical assessment, and screening. The CFI, in comparison, is particularly suitable for engaging in therapeutically meaningful conversations around in-between identities.

To be clear, we are not proposing that the CFI represents a catch-all solution that can singlehandedly resolve issues of inadequate cultural sensitivity, humility, and safety in mental healthcare. Nor do we imply that full experiential transparency and mutual understanding between patient/client and clinician/therapist is always attainable, or even necessarily desirable (141). Moreover, we do not believe that any reluctance to address experiences of in-betweenness and racism in mixed-race patients or clients are generally due to ignorance or individual racist tendencies among mental healthcare professionals. There are, of course, many clinicians and therapists who themselves identify as multiracial or multiethnic and who do not necessarily find it challenging to engage with these issues [although concerns about insufficient racial and ethnic representation among psychotherapists and other mental healthcare professions have been raised in many settings (142–144)]. Moreover, practical issues such as time constraints, limited resources, and competing clinical demands may very well constitute more pressing obstacles—even though it should be noted that such challenges can themselves contribute to systemic-level racial disparities and the “racism without racists” that sociologist Eduardo Bonilla-Silva has discussed (145). In our experience, those clinicians and therapists who do find it difficult to discuss race and racism usually want to develop greater confidence and competence in addressing these issues. Here, the CFI can provide a stepping stone and serve as a catalyst for learning and professional development. Consistent with other research and clinical experience, we believe that the CFI should be used flexibly, guided by situational judgment, and in a manner that allows for non-formulaic phrasing and follow-up questions, so as to avoid unintentionally perpetuating stigma related to mental illness or race and ethnicity (146).

## 8 Conclusion

The number of multiracial families are steadily growing across the globe, and Sweden is no exception. These demographic shifts challenge traditional racial categories and raise important questions for mental healthcare, particularly in supporting individuals navigating increasingly complex racial and ethnic identities. This article has outlined key issues related to the mental health of mixed individuals, including transracial adoptees, and introduced the CFI as a valuable instrument for addressing their needs in clinical practice. By emphasizing a person-centered approach that recognizes fluid and in-between identities, we hope to help practitioners better respond to the diverse experiences within multiracial families. We suggest that the CFI can be highly useful in addressing important aspects of mixed identity and the complexities of navigating a racialized world as a multiracial individual—topics that too often remain unspoken or overlooked in clinical practice. Ultimately, we hope that this article will encourage clinicians to gain both skills and courage to engage more deeply with the lived experiences of multiracial and multiethnic individuals, including transracial adoptees, and to be able to approach identity as a layered, dynamic and relational process. By fostering culturally attuned and inclusive practices, mental healthcare has the potential to better reflect the realities of an increasingly diverse society.

## Data Availability

The original contributions presented in the study are included in the article/supplementary material. Further inquiries can be directed to the corresponding author.

## Footnotes

1 In this article, we have chosen to capitalize both Black and White to indicate that we are referring to social categories rather than to color per se. Whereas this is commonplace when referring to Black people, it is still somewhat more unusual to capitalize White. However, although White people may less often think of themselves in terms of shared racially-based identity, we believe that it is reasonable to emphasize the fact that Black and White are both historically created racial categories and not exclusively treat Whiteness as a naturally existing entity. To be clear, Whiteness here refers to a socially constructed and historically porous position which functions as a normative ideal, defining what is considered neutral, desirable, or fully human, while positioning non-White groups as “Other”.

2 Given Sweden’s longstanding history of high-quality, publicly available population data through the Statistics Sweden agency, the fact that statistics describing the multiracial Swedish population have thus far only been made available as commissioned data for a feature piece in the tabloid newspaper *Aftonbladet* may be seen as indicative of multiraciality as being a blind spot in Swedish research. Using publicly available data from the Statistics Sweden database, and taking the number of individuals with one Swedish-born and one foreign-born parent as a proxy for multiraciality, we estimate that roughly 8.7 percent of the approximately 1.6 million Swedes aged 18–30 have a mixed-race background.

3 A few additional undergraduate papers on this topic exist. Moreover, researchers such as Tobias Hübinette, Daphne Arbouz, Ionna Blasko, and Nikolay Zakharov have explored aspects of multiraciality in Sweden that do not primarily concern identity processes and emotional well-being.
